# Heterozygous Mutations of PTEN in Macrocephaly Patient With Epilepsy: A Case Report

**DOI:** 10.1155/carm/5190615

**Published:** 2025-07-25

**Authors:** Lan Wang, Yilin Su, Mingshu Mo

**Affiliations:** Department of Neurology, The First Affiliated Hospital of Guangzhou Medical University, Guangzhou, China

**Keywords:** epilepsy, heterozygous, macrocephaly, missense mutation, PTEN

## Abstract

Phosphatase and tensin homolog (PTEN), a tumor suppressor gene, is also associated with neurological phenotypes, including macrocephaly, Cowden syndrome, and autism spectrum disorder. We present a 34-year-old Chinese male who complained of recurrent seizures within one year. His occipital frontal circumference was 62.8 cm. Whole-exon sequencing revealed that he carried a heterozygous missense mutation of NM_000314.4:c.4375C > T (p.Met35Val) in PTEN gene. Therefore, heterozygous mutations of c.103A > G in PTEN may increase the risk of macrocephaly with epilepsy.

## 1. Introduction

Macrocephaly (OMIM: 153470) is defined as an abnormally large head circumference, referred to as an occipital frontal circumference greater than 2 standard deviations of the mean [1]. Developmental anomalies of the scalp, cranial bone, and intracranial tissues may contribute to enlarged head circumference [2]. Macrocephaly has nonsyndromic and syndromic classifications divided by physical or behavioral abnormalities [2]. More detailed categorizations are based on the characteristics of physical, metabolic, and brain imaging findings, including familial macrocephaly, autism spectrum disorders (ASD), phosphatase and tensin homolog (PTEN) hamartoma tumor syndrome (PHTS, OMIM: 158350), and others [3–5].

PTEN, located at chromosome 10q23, was identified as a tumor suppressor gene, and its germline mutations induced autosomal dominant tumor syndromes, defined as PHTS (OMIM: 158350) [4]. In the central nervous system (CNS), PTEN protein can function as a lipid phosphatase to antagonize the PI3K/Akt/mTOR pathway and regulate neurological development and maintenance [6]. PTEN mutations reportedly increase the risk of ASD and macrocephaly [7]. The symptoms of ASD include sensory hypersensitivity, impaired social behavior, restricted interest, repetitive behavior, and others. A retrospective study showed that 50% of patients who were diagnosed with ASD had known PTEN mutations, while 66% had macrocephaly [8]. PTEN mutations were more common in patients with both ASD and macrocephaly symptoms [8]. Here, we report a patient with macrocephaly with epilepsy but not ASD who carried a missense mutation of NM_000314.4:c.4375C > T (p.Met35Val; RCV001254105.6) in PTEN gene, which should help to reveal a special genotype–phenotype correlation on PTEN.

## 2. Case Description

A 34-year-old Chinese male visited our neurology department for recurrent seizures. The first symptom was found at the patient's age of 33 while he was typing in front of the computer. His colleague reported that he had sudden rhythmic body and limb jerking for a few seconds, accompanied by loss of consciousness. Later, he developed muscle rigidity with eyeballs rolling upward for nearly 5 min before relief. He did not experience foaming at the mouth, incontinence in urination or defecation, limb numbness, visual ghosting, sweating, dizziness, headache, nausea, vomiting, or other complaints. After relief of seizures, he could not remember the progression of symptoms. He recovered without any sequelae after no treatments. After nearly 1 year, he went to our clinic, he had a similar body and limb jerking again at dinner. The symptoms persisted for nearly 5 min and then resolved.

**Figure 1:**
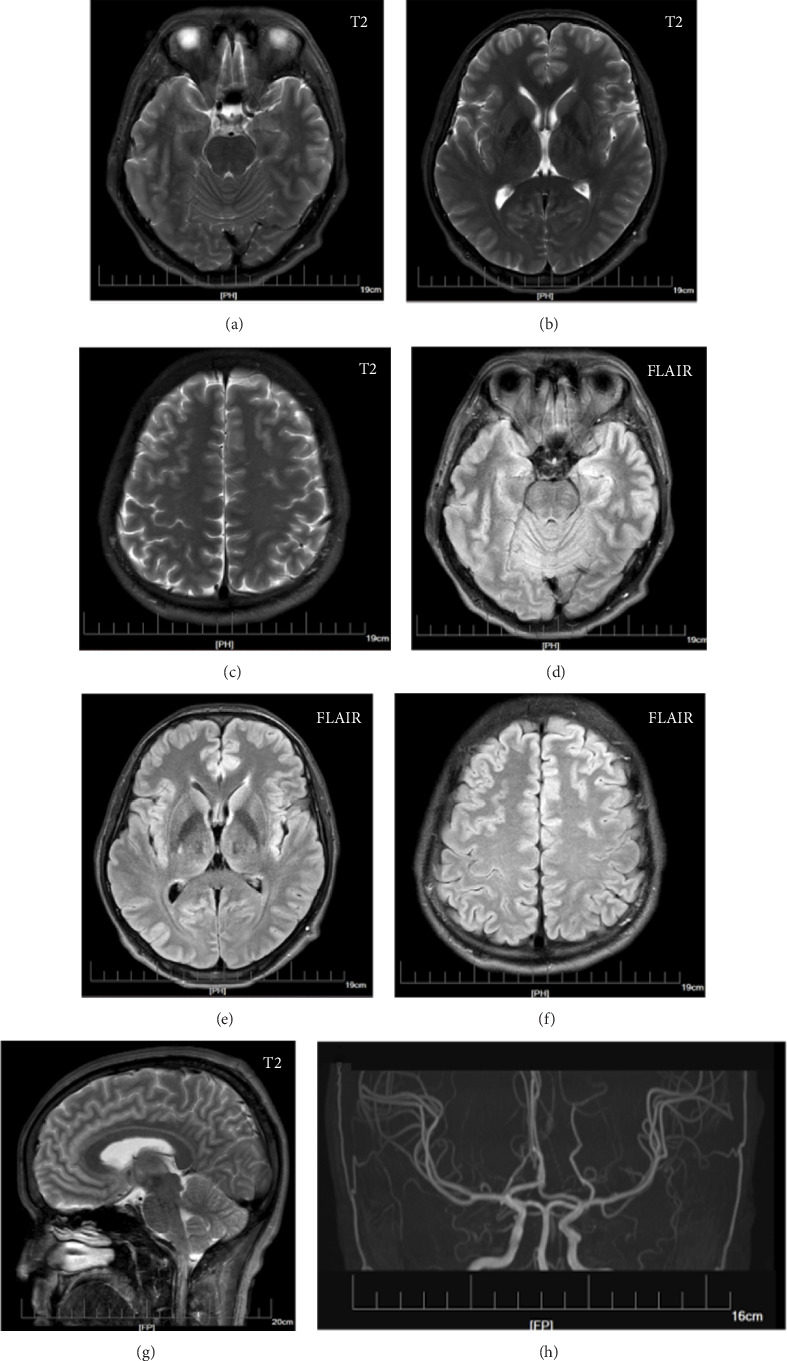
MRI and MRA images of a patient with macrocephaly. Axial T2WI (a–c), FLAIR (d–f) and sagittal T2WI (g) images of different patient cross-sections are shown. The cerebrovascular features were detected by MRA (h).

**Figure 2:**
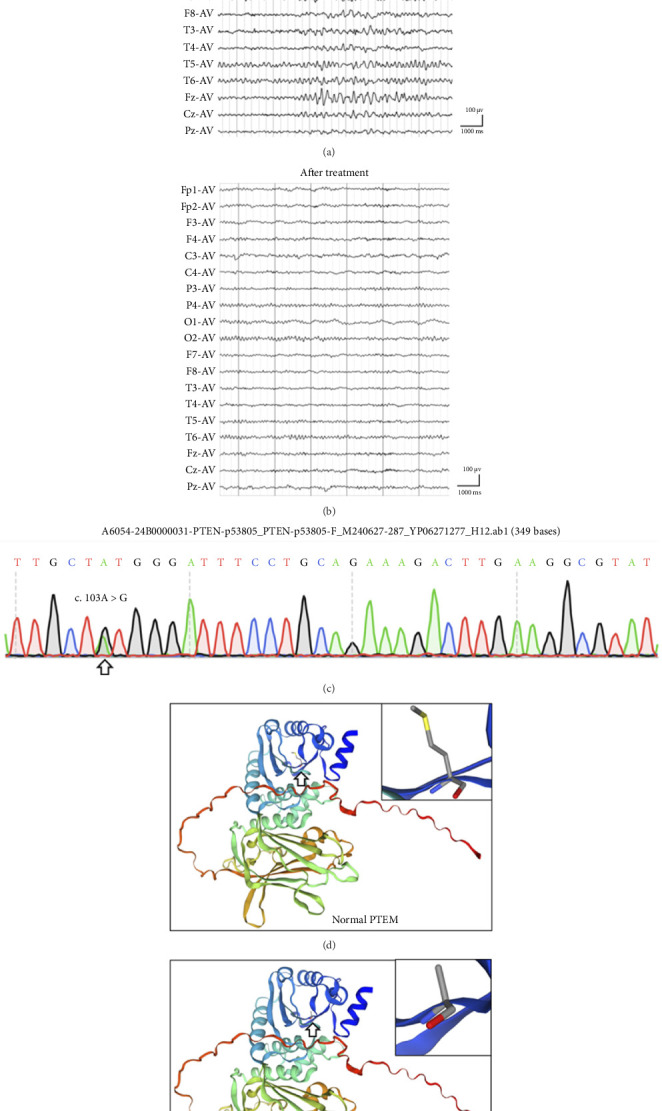
DNA sequencing and EEG of the macrocephaly patient. EMG was performed before (a) and after (b) oxcarbazepine treatment for 3 months. Whole-exon sequencing revealed that the patient carried the c.103A > G mutation in the PTEN gene (c). The 3D protein structure of PTEN was predicted under normal conditions (d), and the p.Met35Val mutation (e) by the Swiss model (https://swissmodel.expasy.org).

He was the only child of healthy Chinese parents and was born via eutocia. His parents and grandparents had no history of tumors, epilepsy, ASD, PTHS, and similar symptoms. He finished secondary education with a passing grade and then worked as a clerk. He had no history of prior hospitalization. The general physical examination revealed that his occipital frontal circumference was 62.8 cm. The cognitive examination tests of attention/orientation, memory, language, visual perceptual, visuospatial skills, and executive function were normal. And the psychiatric assessments were normal, with seven on the Hamilton Rating Scale for Depression (HAMD) and six on the Hamilton Anxiety Scale (HAMA) [9]. The neurological examination was normal. His brain MRI, including T2-weighted imaging (T2WI), fluid-attenuated inversion recovery (FLAIR), imaging and magnetic resonance angiography (MRA), revealed an enlarged head circumference without other apparent abnormalities, which were mainly induced by the large brain parenchyma (Figure 1). The electroencephalogram (EEG) was abnormal, and some recurrent *θ* waves of 20–90 μV and 4–6 Hz emerged in the bilateral cerebral hemispheres, while the partial waveform sharpened (Figure 2(a)). He was diagnosed with unknown epilepsy, received oxcarbazepine treatment and then did not experience any subsequent epileptic episode. Three months later, the patient underwent EEG test again, and the results were normal (Figure 2(b)).

Exome sequencing (MGISEQ-2000 platform) in peripheral blood revealed that he carried a novel heterozygous missense mutation of c.103A > G in PTEN [10]. The Gene mutation of c.103A > G was confirmed by Sanger sequencing, and predicted to induce p.Met35Val in the protein (Figure 2(c)). The carrier frequency of c.103A > G was 0.00 in the normal population based on the ESP6500, ExAC, GnomAD, and GnomAD-EAS databases, and the frequency of G was 0.000004 (1/264690) in trans-omics for precision medicine (TOPMED) and 0.00 (0/10680) in allele frequency aggregator (ALFA) databases. The SIFT, mutation tester, Condel, and Splice AI bioinformatics software programs all predicted that c.103A > G mutation was D; D; deleterious; 0.8. Conservative nucleic acid predictions were performed, and PhyloP Vertebrates, GERP++, PhyloP all suggested that c.103A > G mutation was conserved. The c.103A > G mutation was classified to pathogenic following the American College of Medical Genetics and Genomics (ACMG) 2015 criteria. The prediction also showed that p.Met35Val replacement had little effect on the 3D protein structure of PTEN according to the Swiss model (Figures 2(d) and 2(e)). Published studies about c.103A > G in PTEN gene on PubMed and Google Scholar have reported two cases related to PHTS and Cowden syndrome (OMIM: 158350) and reported as pathogenic in ClinVar [11–13].

## 3. Discussion

Abnormally enlarged intracranial contents are a main cause of macrocephaly, which mostly refers to disorders of neurological development. Epilepsy and cognitive impairment are common neurological symptoms after maldevelopment of the CNS [14]. Macrocephaly, epilepsy and cognitive impairment share a similar mechanism of genetic mutation [4]. Here, we present a case of 34-year-old Chinese male characterized by macrocephaly and recurrent seizures without severe cognitive problems. He carried a heterozygous missense mutation of c.103A > G in exon two of PTEN gene. The transcript variant of this gene is NM_000314.4, and its variant causes the amino acid to change from methionine (Met) to valine (Val). A related review revealed that the c.103A > G mutation in PTEN is associated with Cowden syndrome [15]. This case may help to explore multiple symptoms related to PTEN mutation in neurological disease.

Macrocephaly is classified as a disorder of neurological development related to multiple factors [13]. In addition to cranial hyperostosis and hydrocephalus, megalencephaly of the large brain is a common subtype [1]. PTEN is a key factor in brain development [4]. PTEN dysfunction may induce enlargement of the cerebellum, ventricles, and white matter but has limited effects on the cortex [13, 16]. White matter lesions can induce general anxiety, developmental delay, cognitive impairment, epilepsy, and other psychiatric disorders [17]. Mutations in PTEN may cause symptoms of ASD, but the main problem of patients in this case was epilepsy [8]. A transgenic mouse study revealed that PTEN dysfunction can cause recurrent seizures with an enlarged corpus callosum, which may be caused by increased synaptic connectivity and excitatory amplitude [18]. This kind of abnormally enlarged white matter is found to have more glia with increased proliferative ability [18]. PTEN is a well-known tumor suppressor gene that regulates cell growth and proliferation, possibly through glial proliferation, in patients with macrocephaly, especially in astrocytes and oligodendrocytes [4]. On the other hand, lipids are the largest component of dry brain tissue. PTEN has another biological function in regulating triglyceride accumulation and lipid phosphatase activity [9]. Dysfunction of PTEN may impair lipid metabolism in the brain, resulting in enlarged white matter and epilepsy [9]. In this case, the patient had macrocephaly and an epileptic episode, and these special symptoms may have been caused by a mutation in PTEN.

PTEN is involved in the regulation of cell survival, proliferation, migration, metabolism, and genomic stability [4]. The PTEN gene is located on chromosome 10 and contains 9 exons, and its protein contains 5 key domains, including an N-terminal PIP2-binding domain, a catalytic phosphatase domain (amino acids 7–185), a C-terminal tail, and other domains [19]. In this case, the c.103A > G mutation is located in exon 2 and induces the replacement of Asp with Val in the catalytic phosphatase domain. Similar to the impact of the G129E and Y138L mutations, c.103A > G may also impair the lipid or protein phosphatase activities of PTEN [4]. The phosphatase function of PTEN regulates the substrate of lipid membrane PIP3 and the related PI3K-AKT-mTOR pathway, which are involved in cell proliferation and other biological functions [6]. Additionally, PTEN can dephosphorylate its residues and other signaling molecules, such as cAMP-responsive element-binding protein 1 (CREB1) and insulin receptor substrate 1 (IRS1) [20]. Thus, it is suggested that the c.103A > G mutation can suppress the function and activity of PTEN. PTEN is known to act as a potent tumor suppressor, and subtle decreases in its expression and activity may increase the susceptibility and progression of cancer [13]. However, the role of the function and activity of PTEN in neurological disease is still unclear. Here, we report a mutation, c.103A > G of PTEN, in a patient with epilepsy and macrocephaly, which suggests that the activity of PTEN plays a role in the pathogenesis of these symptoms. On the other hand, p.Met35Val replacement is predicted to have a limited effect on the structure of the PTEN protein, which may be related to mild symptoms in this patient. In this study, due to the parents' refusal of genetic testing, we were unable to determine the inheritance pattern of this specific mutation associated with macrocephaly and epilepsy.

In conclusion, macrocephaly and epilepsy are both complicated genetic diseases involving neurological development. The c.103A > G mutation in PTEN may affect its function in brain development. Recent studies and our case suggested that PTEN plays a key role in neurological development and diseases, especially ASD, epilepsy, macrocephaly, and cognitive impairment. Thus, altering PTEN protein levels and activity may be a novel strategy for developing targeted therapies for neurodevelopmental disorders in the future.

## Data Availability

The data that support the findings of this study are available on request from the corresponding author. The data are not publicly available due to privacy or ethical restrictions.
